# Integration of multi-level semantics in PTMs with an attention model for question matching

**DOI:** 10.1371/journal.pone.0305772

**Published:** 2024-08-29

**Authors:** Zheng Ye, Linwei Che, Jun Ge, Jun Qin, Jing Liu

**Affiliations:** 1 College of Computer Science & Information Physics Fusion Intelligent Computing Key Laboratory of the National Ethnic Affairs Commission, South-Central Minzu University, Wuhan, Hubei, China; 2 College of International Business and Economics, Wuhan Textile University, Wuhan, Hubei, China; Huazhong University of Science and Technology, CHINA

## Abstract

The task of question matching/retrieval focuses on determining whether two questions are semantically equivalent. It has garnered significant attention in the field of natural language processing (NLP) due to its commercial value. While neural network models have made great strides and achieved human-level accuracy, they still face challenges when handling complex scenarios. In this paper, we delve into the utilization of different specializations encoded in different layers of large-scale pre-trained language models (PTMs). We propose a novel attention-based model called ERNIE-ATT that effectively integrates the diverse levels of semantics acquired by PTMs, thereby enhancing robustness. Experimental evaluations on two challenging datasets showcase the superior performance of our proposed model. It outperforms not only traditional models that do not use PTMs but also exhibits a significant improvement over strong PTM-based models. These findings demonstrate the effectiveness of our approach in enhancing the robustness of question matching/retrieval systems.

## Introduction

Question matching is a task of Semantic Text Similarity (STS), which aims to measure the semantic equivalence of two questions. In other words, it determines if two questions are talking about the same thing. This task has high commercial value and plays an important role in a number of fields such as information retrieval [1], short text classification, and customer service [2]. Normally, the questions are short and somehow hard to extract features for machine learning models due to the diversity of word usage and the flexibility of sentence structure, which make this task hard to resolve [3].

In recent years, it has attracted much attention [4]. Traditionally, the TF-IDF (Term Frequency-Inverse Document Frequency) weighting metric is used to extract features. Then a machine learning model is applied to determine the similarity between two sentences. However, TF-IDF features of words cannot capture the word diversities and intrinsic structure of sentences, which are critical for the semantics of a sentence. With the development of deep learning, a number of deep-based approaches have been proposed and shown to be effective for question matching tasks in recent years and bringing a remarkable revolution in the industry. Technically, a variety of neural architectures have been used for this task, which includes Multilayer Perceptron (MLP), Convolutional Neural Network (CNN), Recurrent Neural Network (RNN), Graph Neural Network (GNN) [5] and attention-based model [6]. The neural-based networks have the advantages of automatically extracting features, integrating diversified features, and being trained end-to-end. These models can be grouped into two categories, which are 1) “Siamese network”, and 2) “matching-aggregations” [7, 8]. The first one encodes the two questions separately using the network with shared parameters, and then a similarity function (e.g. cosine similarity) is used for measuring. The benefit of this framework is not only lowering the complexity in network training but also significantly improving the online retrieval performance since the representations of candidate questions can be computed offline. However, the interaction between two questions can not be captured in the framework of the “Siamese network”. Instead, the “matching-aggregations” framework uses attention mechanisms and aggregates corresponding features into one vector, and then a classification head is used to determine the similarity of two questions. Aggregation-based models usually achieve better performance though with lower efficiency. In this paper, we also follow this aggregation strategy.

In addition, with the rapid development of Large-scale pre-trained models (PTMs) such as BERT [9], GPT [10], and ERNIE [11], the paradigm of NLP has changed somehow. Since large-scale PTMs can effectively capture knowledge from massive labeled and unlabeled data, the fine-tune strategy with a task-specific head on top of PTMs achieves great success and sets new SOTAs on a large number of NLP tasks [11]. Normally, only the last layer of the PTM models has utilized for the final task-specific prediction. However, researchers [12] found that the representations learnt by BERT have a nature of capturing rich hierarchy linguistic features, with surface features in lower layers, syntactic features in middle layers and semantic features in higher layers. They conducted a series experiments with ten probing sentence-level datasets/tasks [13] to probe this nature, and evaluated the performance of every BERT layers. The results show that the surface tasks tended to have best scores at bottom layers, the syntactic tasks outperformed in middle layers while the semantic tasks at the top. Inspired by [12], we combined features learned from different layers in PTMs to conquer the challenge with complex structure and word diversity in question matching. In particular, we employ the attention mechanism to make different layers pay more attention to a different category of question pairs.

The main contributions can be summarized as follows: 1) we propose a novel attention-based matching model called ERNIE-ATT, which integrates multi-level semantics in different layers of PTMs. 2) extensive experiments on two challenging datasets show the effectiveness and robustness of the proposed model. We also visualize the attention of different layers for better understanding.

The remainder of this paper is organized as follows: in the section Related Work, we review the development and current status of question matching tasks, PTMs and attention mechanisms, and point out their shortcomings. In the section Approach, we present our proposed model. In the section Experiments, we introduce the settings of the experiments, and the experimental results are presented and discussed. Finally, we conclude our work briefly and present future research directions in the section Conclusions and Future Work.

## Related work

### Question matching task

The question matching task is one of the four major tasks (Named Entity Recognition, Question Answering, Language Modeling) of NLP, which mainly refers to the semantic correlation of two sentences. The research and landing application scenarios are prevalent and extensive. Notably, OpenAI has trained ChatGPT and GPT-4 [14] using unprecedented computational scale and data size, which show more general intelligence than previous AI models, and there is a strong correlation between question matching tasks and such large-scale language models (LLM).

Traditional schemes mainly use, for example, TF-IDF, Levenshtein distance, Simhash [15], BM25 [16] and other statistical-based methods to calculate the literal similarity of two texts by lexical overlap. However these are not appropriate for question matching tasks, because these methods mainly considered discrete features. Also complex structure and word diversity, such as symmetry/asymmetry structures and antonyms/synonyms, may linguistically perturb semantic meaning in question pairs [3]. For a simple example, “我喜欢吃苹果”(“I like to eat apples”) and “我不喜欢吃苹果”(“I don’t like to eat apples”). The two sentences have the most literal similarity, but only with a negation, their meanings are opposite.

Nowadays, various neural network models are used for question matching tasks, such as Convolutional Neural Networks (CNNs), Recurrent Neural Networks (RNNs), BiLSTM [17], Text-CNN [18], BiMPM [19], and attention mechanisms. while NLP tasks often rely heavily on discrete manual features, neural network model approaches typically use low-dimensional and dense vectors (aka distributed representations) to implicitly represent the syntactic or semantic features of a language. These representations are learned in the context of a specific NLP task, so neural network modeling approaches allow researchers to solve various NLP tasks more easily.

Despite the success of neural network models in various types of NLP tasks, the performance improvements may be less dramatic compared to the field of computer vision. The main reason is that the current datasets used for most supervised NLP tasks are quite small. Deep neural networks usually have a large number of parameters, leading them potentially overfit to small training data and generalize poorly in real-world applications.

### Pre-trained models

With the advent of the Transformer architecture, deep pre-trained language models (PTMs) employing Transformer, such as BERT (Bidirectional Encoder Representations from Transformers) [9] and GPT (Generative Pre-trained Transformer) [10], have garnered significant success in recent times. The transformative impact of these models is evident through their ability to capture rich linguistic knowledge, achieved by fine-tuning large-scale pre-trained models with extensive datasets. This linguistic prowess translates into exceptional performance on downstream Natural Language Processing (NLP) tasks, surpassing even human performance in certain instances. Consequently, large-scale pre-trained models have become a focal point of intense research interest.

In the training paradigm of widely adopted BERT models, training is conducted at the token level. In contrast, Baidu’s ERNIE (Enhanced Representation through kNowledge Integration) [20] takes a more comprehensive approach by incorporating three levels of training: token level, entity level, and phrase level. This nuanced strategy enables ERNIE not only to capture token information but also to glean more profound insights from the semantics embedded in the preceding and following texts.

The latest iteration, ERNIE 3.0 [11], introduces an innovative fusion of autoregressive and self-coding networks for pre-training. The self-coding network leverages ERNIE 2.0 [21]’s multi-task learning, incrementally constructing pre-training tasks. This unique framework amalgamates big data pre-training with knowledge derived from multiple sources. ERNIE’s continuous learning techniques facilitate the absorption and acquisition of lexical, structural, and semantic knowledge from vast textual data, resulting in the continual evolution of model effectiveness. Across 16 public datasets encompassing text or question matching, intelligent QA tasks, sentiment analysis, natural language inference, lexical analysis, and reading comprehension, ERNIE establishes state-of-the-art (SOTA) performance.

While the accuracy of these PTMs rivals or even surpasses human performance across various NLP tasks, they exhibit limitations in robustness when confronted with complex scenarios. Addressing this research imperative is crucial to enhancing the practical utility of these models and advancing the development of semantic matching techniques.

### Attention mechanisms

Attention mechanisms have arguably become one of the most important concepts in the field of deep learning. With the development of deep neural networks, the attention mechanism can be used as a resource allocation scheme and is a major means of solving the information overload problem. It can process more important information with limited computational resources.

In the field of NLP, the computation of attention, in general, is divided into two main steps: the first step is to compute the attention distribution over all input information, and the second step is to compute a weighted sum of input information based on the attention distribution, by which key information is selected. In addition, there are other variants of attention mechanisms, such as Hard Attention, which focuses on only one input vector, Multi-head Attention, which focuses on multiple parts, and Self-attention, which uses the information inherent in the features to interact with attention (e.g., Transformer), etc.

Nowadays, work on the interpretability of large PTMs like BERT [9] and ERNIE [11] has become a trendy direction in NLP. This could not only help us understand the reasons behind the superior performance of PTMs but also its limitations, which can guide the design of improved model architectures. It has been shown that when such PTMs are evaluated for their ability to track subject-predicate agreement, it captures syntactic phenomena well. It has also been shown that the middle layers within the model encode a rich hierarchy of linguistic information, with surface features in the lower layers, syntactic features in the middle layers, and semantic features in the higher layers [12].

Based on the study of the internal structure of the PTM, different layers in the PTM have different semantic information, i.e., different layers may be suitable for different categories of problems. In the question matching task, selecting a suitable attention mechanism to guide the integration of different layers of information based on the study of the internal structure of the PTM is a viable direction to address the challenges of the complex structure and word diversity of the question matching task.

## Approach

To establish clarity and coherence throughout this paper, we begin by introducing the notations and conventions employed in our discourse.

Subsequently, we unveil our novel attention-based model, named ERNIE-ATT. This model is intricately designed to leverage attention mechanisms for enhanced performance and will be elaborated upon in the following sections.

### Problem formulation

In the context of question matching, we are presented with two input sentences constituting a question: *Q* = [*q*_1_, …, *q*_*i*_, ‥, *q*_*m*_], where *m* represents the length, and *T* = [*t*_1_, …, *t*_*i*_, ‥, *t*_*n*_], with *n* denoting the length. Here, *q*_*i*_ and *t*_*i*_ denote the *i*-th word in the question *Q* and *T*, respectively.

The objective of this task is to define a function *y* = *s*(*Q*, *T*), mapping the question pair (*Q*, *T*) to a binary label in the set {0, 1}, indicating whether the two questions are equivalent or not.

### The proposed model

**Fig 1:**
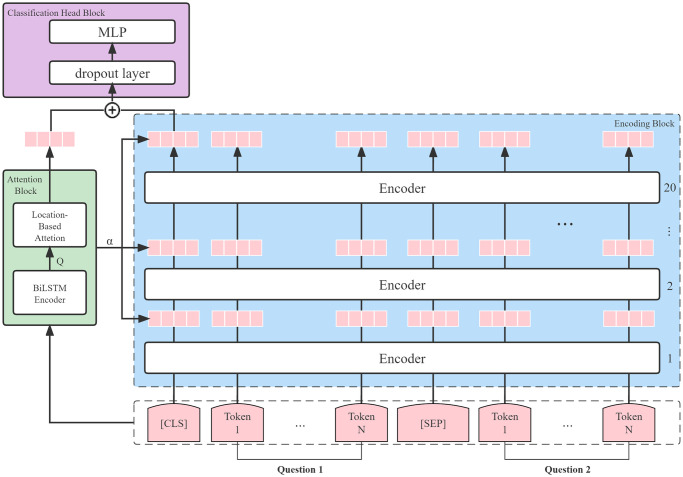
Model architecture. Our revolutionary ERNIE-ATT architecture is structured into three key components: an encoding block for feature extraction, a block dedicated to directing the significance of different layers through a location-based attention mechanism, and a classification header block tailored for the question matching task. (The symbol *α* refers to the weight *α* generated by the attention block for the current question pair, which will be combined with the extracted features of each layer in the encoding block to better know which layer learned features are more important for the current question pair).

The overall structure of our proposed model is illustrated in Fig 1. The model comprises three key components, outlined as follows:

1. **Encoding Block with PTMs for Feature Extraction:** The initial building block involves the encoding phase utilizing Pre-trained Models (PTMs) to extract features. Each layer of PTMs contributes fundamental features for subsequent processing.
2. **Attention Block for Guided Feature Importance:** The attention block plays a crucial role in determining the significance of different layers concerning the question pair. It integrates the features obtained in the encoding phase, providing a guided focus on relevant information.
3. **Classification Head Block for Question Matching:** The final building block is dedicated to the question matching task. This block encompasses the classification head, responsible for making distinctions between question pairs based on the extracted and attentively guided features.

In the subsequent sections, we delve into a comprehensive exploration of each building block, elucidating their respective functionalities.

#### Encoding block

We use pre-trained language models (PTMs) for input encoding. Specifically, ERNIE [11] is used in our experiments, which was trained with 10 billion parameters on a 4TB corpus consisting of plain texts and a large-scale knowledge graph.

ERNIE outperforms state-of-the-art models on 54 Chinese NLP tasks. Thus, the encoding block can be defined as a basic feature extracting function Eq (1), where *N* is the maximal sequence length of the input, *L* is the number of layers of PTM used and *h* is the size of hidden layers.

$$\begin{array}{c} f\left(Q,T\right)\to A^{N\times L\times h} \end{array}$$ (1)

For the task of question matching, we use the off-the-shelf sentence pair classification paradigm described in [22] as shown in the encoding part of Fig 1. The model takes question tokens and title tokens as input, with two special tokens [*SEP*] separating the two segments and [*CLS*] indicating the beginning of the input. Normally, the [*CLS*] embedding in the last layer, which is the first token of the input, is used as a classification token since it contains specific classification information.

#### Attention block

As previously discussed in the Introduction section, the progressive learning of linguistic information across different layers of Pre-trained Models (PTMs) holds promise for addressing the challenges posed by question matching involving complex structures and diverse vocabulary. In this paper, we introduce an attention mechanism designed to effectively integrate information from different layers.

The attention block, illustrated in the attention component of Fig 1, comprises two encoders: (1) Bidirectional LSTM (BiLSTM); (2) Location-based attention. The BiLSTM encoder takes as input the original word embeddings (prior to the first encoding layer) extracted from PTMs, as depicted in Fig 1. Utilizing bidirectional LSTM is essential, given that the positions of input questions are interchangeable. The BiLSTM encoder aggregates information from the question pair, producing a coarse representation that serves as the input for the attention layer.

Various attention mechanisms, including additive attention, location-based attention, and others [23], can be employed. In our experiments, we opt for location-based attention and additive attention due to their superior empirical performance.

Location-based attention, computed according to Eq (2), derives alignment scores exclusively from the target hidden state. Notably, the attention scores are solely linked to the query *q* rather than the key *k* [22], recognizing that different layers in PTMs may excel in handling distinct categories of questions.

$$\begin{array}{c} f(q,k)=f(q) \end{array}$$ (2)

Additive attention is another type of attention mechanism in which the attention score is computed by taking the dot product of the query and key vectors followed by adding a bias term and applying a non-linear activation function. The formula for additive attention can be represented as Eq (3), where *v*, *b*, *W*_1_, *W*_2_ represent the learnable parameters in the model. The activation function, denoted as *act*, is a non-linear function that can be either tanh or ReLU.

$$\begin{array}{c} f\left(q,k\right)=v^{T}act\left(W_{1}k+W_{2}q+b\right) \end{array}$$ (3)

#### Classification head block

To form the final features, we concatenate the output of the attention layer with the pooler output of the *CLS* state from the last layer. Subsequently, a dropout layer is applied on top of this concatenation. For the sake of simplicity and computational efficiency, we utilize a straightforward multiple-layer perceptron (MLP) network with sigmoid activation for the final prediction.

## Experiments

### Datasets

All the experiments are conducted on two diverse Chinese datasets, i.e.,**LCQMC** [24] and **BQ** [25]. Dataset format and model prediction examples are shown in Table 1 below. In addition, the number of equivalent and unequal question pairs in the dataset is almost evenly distributed.

**Table 1:** Dataset format and model prediction examples.

Question 1	Question 2	Label	Prediction	Category
胎儿什么时候临盆 (When will the fetus be born)	胚胎什么时候临盆 (When will the embryo be born)	1	1 or 0	TP or TN
人民币怎么换港币 (How to exchange RMB for HKD)	港币怎么换人民币 (How to exchange HKD for RMB)	0	1 or 0	FP or FN

The training and validation sets are composed of a number of Question1 and Question2 pairs and labels, while the test set is composed of question pairs consisting of Question1 and Question2 only. Our task is to determine whether each question pair is semantically matched or equivalent. The only possible prediction of the question matching model for each question pair is 1 or 0. We use the prediction result to compare with the label to determine whether it is TP, TN, FP, or FN, and the ACC evaluation metric is based on them to calculate the proportion of the number of correct classifications in the total number of samples. The English below each sentence is the corresponding translation for easier reading.

#### LCQMC corpus

It is a large-scale Chinese QM dataset that focuses more on intent matching rather than paraphrasing. It contains 260,068 question pairs and splits it into three parts, i.e., the training set consists of 238,766 question pairs, the development set consists of 8,802 question pairs and the test set contains 12,500 question pairs. The construction method is to extract high-frequency related questions from Baidu Knows from various fields, and then execute preliminary filtering by Wasserstein Distance, which is based on the algorithm called word mover distance (WMD), and finally manually label them. Positive samples are 30% more than negative samples.

There must be a large number of repetitive questions in these questions, and the repetitive questions are expressed in various forms, which provides the possibility of constructing large-scale sentence pairs for question matching. According to the experimental results of the original paper comparing unsupervised and supervised methods, there is an absolute improvement of about 10% on average in both F1 score and ACC, and an improvement of about 15% on average in precision [24]. This demonstrates the effectiveness of not only the supervised method but also the proposed LCQMC corpus.

#### BQ corpus

It is the first financial-domain Chinese QM dataset for sentence semantic equivalence identification (SSEI), which consists of 120,000 question pairs from online bank custom service records. It is split into three parts with no overlap: 100,000 pairs for training, 10,000 pairs for validation, and 10,000 pairs for testing. The number of positive and negative samples is the same.

Firstly they used methods of grouping and clustering to get distinct groups and questions for annotation. Then, annotators clustered questions into different intentions and distinguish them. Finally, they selected positive or negative questions to generate positive or negative question pairs in distinct intent stacks.

The original data was obtained from a 1-year customer service log provided by a Chinese bank, which contains over 20 million questions. The lower performance on the BQ corpus compared to the performance on the Quora corpus suggests that the BQ corpus is challenging to research on semantic matching models and is well suited for use as a training and testing of question matching models.

#### Test sets

We would like to mention here in particular the construction of the test sets. Most current question matching tasks use a single metric to assess how well a model does on homogeneously distributed test sets, which may exaggerate the model’s capabilities and lacks a fine-grained assessment of the model’s robustness in terms of strengths and weaknesses. We chose the test sets of LCQMC and BQ, which can focus on the robustness of the question matching model in real application scenarios, and evaluate the model’s performance from three major dimensions: lexical, syntactic, and pragmatic, expecting to evaluate the model’s performance from multiple dimensions and domains, so as to objectively and accurately evaluate the model’s performance.

### Evaluation metric

We evaluate the performance of each model using the mainstream classification task evaluation method, **accuracy (ACC)**, which uses TP (True Positive), TN (True Negative), FP (False Positive), and FN (False Negative) to calculate the proportion of correctly classified samples among the total number of predicted samples. Since there is no way to evaluate model performance based on precision and recall directly, we also introduce the **F1-score** metric, which can make a balance between precision and recall to achieve the best performance.

$$\begin{array}{c} ACC=\frac{TP+TN}{TP+TN+FP+FN} \end{array}$$ (4)

$$\begin{array}{c} Precision=\frac{TP}{TP+FP} \end{array}$$ (5)

$$\begin{array}{c} Recall=\frac{TP}{TP+FN} \end{array}$$ (6)

$$\begin{array}{c} F1=2\cdot \frac{Precision\cdot Recall}{Precision+Recall} \end{array}$$ (7)

### Baseline models

In the experiments, we mainly compare our model with a number of popular baselines, including the state-of-the-art ERNIE-based PTM model, which achieves SOTA performance on a number of downstream NLP tasks. We do not report results of traditional retrieval-based baselines (e.g. BM25 [16]) since it performs pretty badly in this scenario.

The following section provides concise descriptions of the eight baselines considered in our comparative analysis.

#### Text-CNN

It is a classical Convolutional Neural Network (CNN) text classification model [18]. It is trained by a simple CNN with one layer of convolution on the word vector of an unsupervised neural language model. Although the hyperparameters are rarely tuned, this simple model obtains excellent results on several benchmarks, suggesting that the pre-trained vectors are general-purpose feature extractors that can be used for a variety of classification tasks. For example, in the experiments of the BQ dataset paper, they specifically introduced the Text-CNN method to optimize against question matching and obtained excellent results by separately feeding each sentence in the question pair into a model with a 300-dimensional word vector and stitching the sentence representation into the SSEI.

#### BiLSTM

[17] It is an extension of the RNN (Recurrent Neural Network) model, which consists of a forward LSTM and a backward LSTM. They use the same structure, but the model composed with BiLSTM can be better optimized for the specific problem of the question matching task. In their experiments, the results show that the BiLSTM model is able to learn the dependency features between words better than other models.

#### BiMPM

It is a model [19] which adopts bidirectional multi-angle matching, not only considering one dimension but also adopting a sarging-aggregation structure to calculate the similarity between two sentences. Given a question pair, they first use a BiLSTM encoder to encode them. Next, match the two sentences in two directions, and then use another BiLSTM layer to generate a fixed-size matching vector from the matching results. Finally, get classification results based on the vector through a fully connected layer. They evaluate their model on paraphrase identification, natural language inference, and answer sentence selection tasks, and experimental results on standard benchmark datasets show that their model achieves state-of-the-art performance on all tasks.

#### BERT-Base

It is the official epoch-making Chinese BERT model [9] released by Google. Instead of the traditional unidirectional language model or shallow splicing of two unidirectional language models for pre-training, the brand new masked language model (MLM) is used for pre-training to generate deep bidirectional language representations. It obtained new SOTA results in 11 NLP tasks, including question answering, sentiment analysis, and natural language inference.

#### BERT-wwm-ext

BERT-wwm [26], or Bidirectional Encoder Representations from Transformers with Whole Word Masking, is an improved version of BERT that was developed in 2019. BERT-wwm uses a technique called whole word masking, which means that it masks entire words instead of individual subwords. This helps BERT-wwm to learn more accurate representations of words and phrases, which leads to improved performance on NLP tasks. BERT-wwm-ext [26] is an extended version of BERT-wwm. BERT-wwm-ext uses the same whole word masking technique as BERT-wwm, but it is also trained on a large dataset of external training data and much more training steps. This additional training data helps BERT-wwm-ext to learn more accurate representations of words and phrases, which leads to the next stage of NLP task performance again.

#### PERT/LERT

Both PERT and LERT are SOTA models that have been specifically optimized for text matching tasks in recent years. PERT is an auto-encoding model [27] trained using the Permutation Language Model (PerLM). Instead of the traditional Masked Language Model (MLM) pre-training task, PerLM predicts the original token positions in a shuffled input text. The authors employed full word masking and N-gram masking techniques to enhance the performance of PERT. On the other hand, LERT combines linguistic features (POS, NER, and DEP) generated by LTP and is pre-trained using a masked language model for multiple tasks. LERT [28] also introduces a pre-training strategy that focuses on acquiring basic language knowledge before higher-level knowledge. Extensive experiments conducted on Chinese and English NLU benchmarks demonstrated the effectiveness and sophistication of both models, resulting in significant improvements over the baseline for various tasks.

#### ERNIE-Base

It is based on the ERNIE PTM model [11] with a simple dense classification head. In particular, we use the large model of ERNIE 3.0 [11] with 20 layers (named **ERNIE-3.0-xbase-zh**) for both ERNIE-based and ERNIE-ATT. ERNIE 3.0 is a large-scale knowledge augmentation model pre-training framework, which fuses auto-regressive network and auto-encoding network and is trained with more than 4TB compound corpus and 10 billion parameters. ERNIE 3.0 outperforms existing models on 54 Chinese NLP tasks and is a very strong baseline of PTMs.

### Parameter settings

There are several hyper-parameters to tune in the experiments. To build strong baselines and make fair comparisons, the parameters for different models are optimized in the same way. Specifically, we sweep i) the learning rate over (*lr* ∈ {5e-6, 1e-5, 5e-5}), ii) dropout rate in (*dp* ∈ {0, 0.1, 0.2, 0.3, 0.4}), iii) the number of layers in MLP over {1, 2, 3}). For other parameters that occurred only in the baseline models, we take the reported values from the original papers.

### Experimental results and analysis

Table 2 presents a basic comparison among all models, with the percentage values in parentheses indicating their performance relative to ERNIE-Base for a fair evaluation. The first three models are classical deep matching models, especially BiMPM, which has demonstrated outstanding performance in the field of text matching. The following five models are the current popular Pre-trained Models (PTMs) in the Chinese language domain. It is worth noting that PERT and LERT are the latest PTMs in this field, showcasing advanced capabilities. The values highlighted with underlines denote the best performance within the current group.

**Table 2:** Comparison of different models.

Models	ACC		F1	
	LCQMC	BQ	LCQMC	BQ
Text-CNN	72.80	68.52	75.70	69.17
BiLSTM	76.10	73.51	75.70	69.17
BiMPM	83.30	81.85	84.90	81.75
BERT-Base	85.73	84.50	86.86	84.00
BERT-wwm-ext	85.60	84.70	87.70	83.90
PERT	86.30	84.30	-	-
LERT	86.60	85.10	-	-
ERNIE-Base	87.29	84.36	88.30	84.26
**ERNIE-ATT**	**89.28**(1.99%)*^,+^	**85.34**(0.98%)*^,+^	**89.84**(1.54%)*^,+^	**85.48**(1.22%)*^,+^

Experiments showed that our introduction of an attention mechanism to guide the integration of information from different layers of PTMs is very effective. Top-level PTMs, such as BERTs, ERNIE-Base, PERT, LERT and ERNIE-ATT, which both outperform the performance of traditional deep matching models, also include the strong baseline performed by the BiMPM model designed specifically for short text matching tasks. Our proposed ERNIE-ATT achieves an absolute improvement of 5.98% and 3.49% over BiMPM in ACC performance on the two challenging datasets and still achieves gains of 1.99% and 0.98% over the performance of the popular Chinese large-scale pre-trained model ERNIE-Base. The performance of ERNIE-ATT in the F1 score is also better than the performance of the traditional deep matching models and ERNIE-Base.

In particular, a “*” and a “+” indicate a statistically significant improvement over **BiMPM** and **ERNIE-Base** respectively, according to the Wilcoxon matched-pairs signed-ranks test at the 0.05 level. The bold phase style in a row means that it is the best result.

Overall, this series of top PTMs (BERT-Base, BERT-wwm-ext, PERT, LERT, ERNIE-Base and ERNIE-ATT) outperform traditional deep matching models, including BiMPM which is a strong baseline specifically designed for short text matching tasks. Even though ERNIE-Base only adds a simple MLP head on ERNIE, it still achieves an absolute 3.99% and 2.51% improvement over BiMPM in terms of accuracy. Furthermore, Ernie-Base outperforms the globally popular BERT, as well as the iterative upgrade version of BERT, namely BERT-wwm-ext. It validates the effectiveness of the large PTM of ERNIE, which makes a strong baseline. In addition, even compared with strong ERNIE-Base, we still achieve relative ACC gains of 1.99% and 1.54% on the LCQMC and BQ datasets respectively. It indicates that integrating information from different layers is useful and our attention mechanism works well in this scenario.

**Fig 2:**
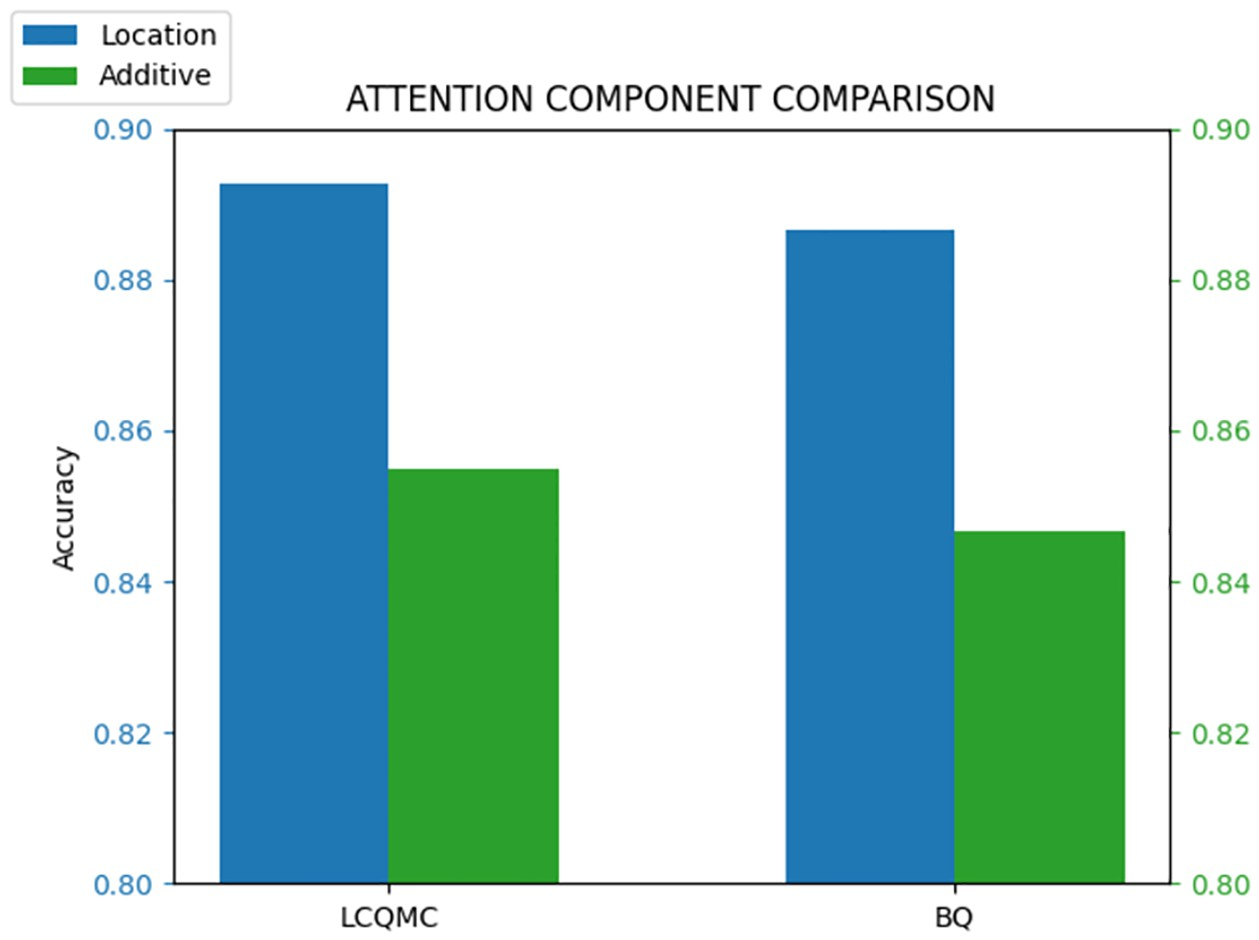
Attention component comparison.

Fig 2 compares the effect of different attention blocks explained in the section Approach. It shows that the location-based attention performs slightly better than additive attention. One possible explanation is that in location-based attention, the attention scores solely depend on the query *q* and not on the key *k*. This characteristic aligns well with the requirement of integrating features from different layers in PTMs. Furthermore, this attention mechanism is easily learnable and demonstrates effectiveness across various question categories.

**Fig 3:**
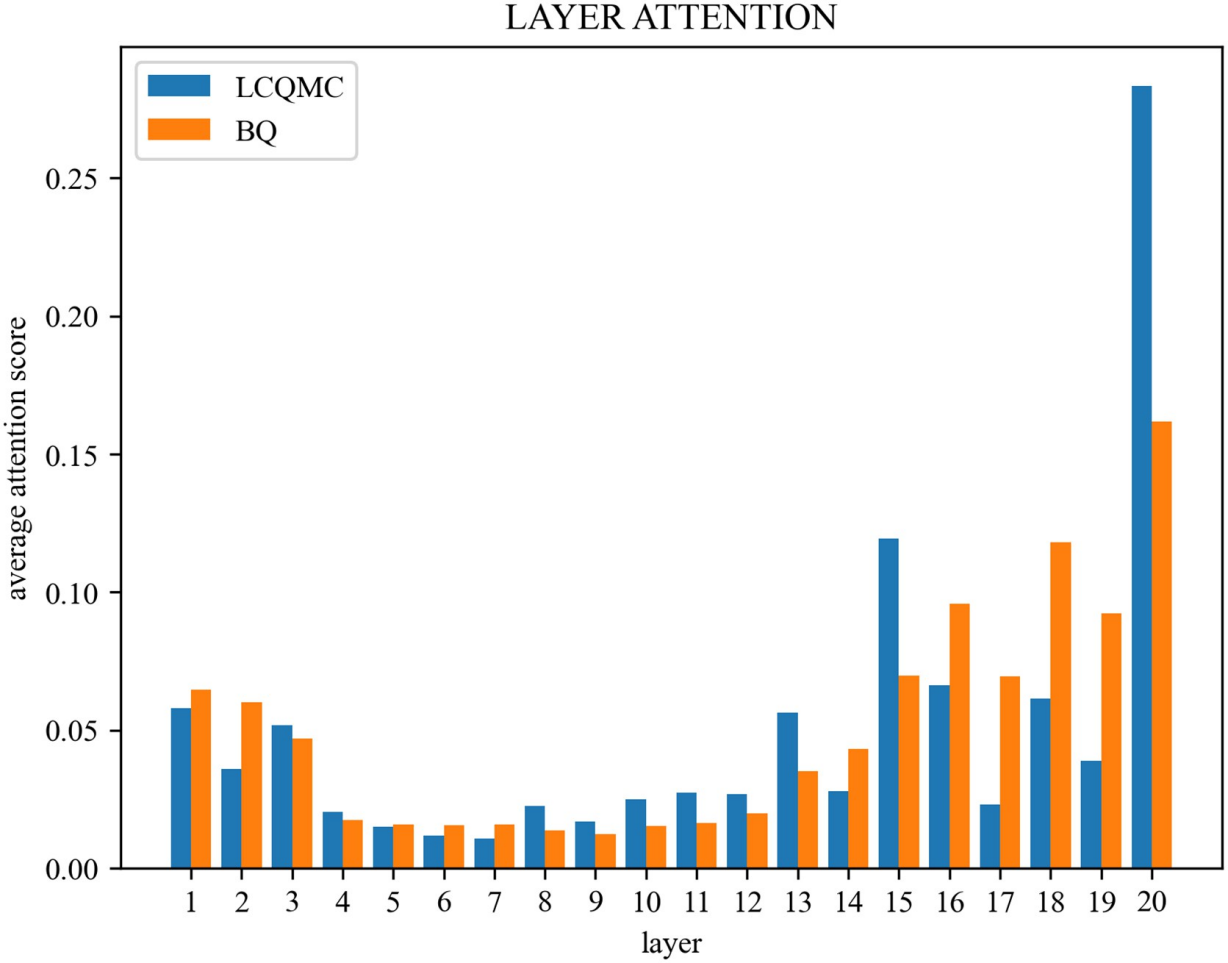
Layer attention. As we expected, ERNIE-ATT not only pays a lot of attention to the higher layers, but our model also gives more attention to the other layers. As the feature learning of PTMs goes deeper, the problem that the original information may be lost to some extent is well resolved on our ERNIE-ATT.

We also visualize the attention weights for each layer as shown in Fig 3. As expected, ERNIE-ATT pays more attention to the higher layers since they contain high-level features for classification. It is also interesting to note that our model also pays much more attention to the lower layers compared to the middle layers. We suspect as the PTMs become deeper, the original information may be lost to some extent.

## Conclusions and future work

In this paper, we propose an attention-based model, called ERNIE-ATT, which is built upon the ERNIE framework. Unlike conventional approaches that solely rely on the last layer for prediction, our model incorporates an attention mechanism to effectively aggregate semantic information learned across all layers of PTM-based models.

Through extensive experimentation on two large-scale datasets, we demonstrate the exceptional effectiveness and robustness of the proposed ERNIE-ATT model. Notably, it surpasses strong baselines of PTMs, including various BERT models and the robust ERNIE-Base, yielding significantly improved performance.

In future work, we aim to further enhance the performance of our ERNIE-ATT model by exploring alternative structures for its three core building blocks. This exploration will enable us to achieve even better results. Additionally, we are interested in investigating the applicability of our model to other NLP tasks, thus expanding its potential impact in the field.
